# Characteristics and clinical outcomes of pulmonary sarcomatoid carcinoma: experience from Tata Memorial Centre

**DOI:** 10.3332/ecancer.2022.1438

**Published:** 2022-08-08

**Authors:** Suresh Kumar Bondili, Ravindra Nandhana, Aditya Dhanawat, Vanita Noronha, Amit Joshi, Vijay Maruti Patil, Nandini Menon, Rajiv Kumar Kaushal, Anuradha Choughule, Sabita S Jiwnani, Amit Janu, Kumar Prabhash

**Affiliations:** Tata Memorial Hospital, Mumbai, Maharashtra, 400 012, India

**Keywords:** non small cell lung cancer, sarcomatoid carcinoma, retrospective analysis

## Abstract

**Background:**

Pulmonary sarcomatoid carcinoma (PSC) constitutes a heterogeneous group of poorly differentiated non-small cell lung cancers. Since these are rare tumours, we sought to determine the characteristics and clinical outcomes of these patients treated at our centre.

**Methods:**

We did a retrospective evaluation of all patients diagnosed with PSC between January 2013 and September 2020 at the Tata Memorial Hospital, Mumbai, India. Baseline demographic and treatment data and outcomes were obtained retrospectively from electronic medical records and survival was calculated by using the Kaplan–Meier method.

**Results:**

Out of 151 patients diagnosed with PSC during this period, 129 were included in the final analysis. The clinical stage was stage I in 3 (2.03%), stage II in 4 (3.1%), stage III in 35 (27.1%) and stage IV in 87 (67.4%). The median follow-up duration was 32 months (range, 15.0–48.9). The median overall survival (OS) of patients who received curative surgery was 18 months (95% confidence interval (95% CI), 2.59–33.4); concurrent chemoradiation was 11 months (95% CI, 2.99–19); palliative chemotherapy was 8 months (95% CI, 5.24–10.75) and best supportive care was 1 month (95% CI, 0.43–1.57, *p* = 0.001). On multivariate analysis, the presence of brain metastasis (p = 0.018; hazard ratio (HR), 2.47; 95% CI, 1.34–4.49) and the administration of chemotherapy (*p* = 0.037; HR, 2.2; 95% CI, 1.04–4.94) were the only factors impacting the OS.

**Conclusion:**

PSC usually presents in advanced stages and is associated with a poor prognosis.

## Introduction

Pulmonary sarcomatoid carcinoma (PSC) encompasses a heterogeneous group of non-small cell lung cancers (NSCLC) with poor differentiation. It is rare and comprises about 0.3%–3% of all lung cancer cases [1].

PSCs are epithelial tumours with a component of sarcomatous differentiation [2]. They include five different histological varieties as per the 2004 World Health Organization (WHO) classification of NSCLC, namely pleomorphic carcinoma, spindle cell carcinoma, giant cell carcinoma, carcinosarcoma and pulmonary blastoma [1, 3].

PSC has a predilection for males and heavy smokers [4]. The usual age at the time of diagnosis is the sixth decade of life. However, pulmonary blastoma occurs with similar prevalence in both men and women and the age of presentation is lower than the other types of PSC and generally occurs in the fourth decade [5, 6]. There are no signs or symptoms which are specific to PSC; they resemble those of conventional NSCLC [7]. Patients with PSC are treated along the lines of NSCLC. Surgery is the mainstay of the management of the early-stage disease [8]. Many retrospective analyses and propensity score analyses have revealed that these patients tend to relapse early and have a worse survival in comparison with patients of similar stage NSCLC [9–12]. PSCs are aggressive, respond poorly to chemotherapy and are less sensitive to radiotherapy. Immune checkpoint inhibitors (ICIs) might have a critical role in the treatment of PSCs as they have high programmed death ligand-1 (PD-L1) scores and remarkable responses to ICIs have been described [13–19]. Moreover, the tumour mutational burden was also found to be high in about 40.6% of patients with PSC in a Chinese study [20].

Because of the complexity involved in the histological categorisation, a well-experienced onco-pathologist is required for an accurate diagnosis. Given the rarity of this disease, no randomised trials are available to guide appropriate clinical management, and the optimal treatment strategies for PSC are not known. Our study aims to evaluate the clinical characteristics, pathological features and outcomes of patients with PSC treated at our centre and may provide important clues for the optimal management of these patients.

## Patients and methods

### Patients

This is a retrospective analysis of patients with PSC diagnosed and treated by the thoracic disease management group of the Department of Medical Oncology, Tata Memorial Hospital, a tertiary cancer centre in Mumbai, India. All patients with pathologically diagnosed PSC between January 2013 and September 2020 and aged > 15 years were identified from the pathology database and included in this study. The cut-off date for survival follow-up was 30 September 2020. The demographic details, comorbidities, family history, histopathological details, radiologic data, treatment administered, response to therapy, follow-up and survival data were obtained retrospectively from the electronic medical records. The study was conducted by the principles laid down by the Declaration of Helsinki and the International Council for Harmonization-Good Clinical Practice.

### Diagnosis and management

The pathological diagnosis was made on the biopsy specimens or after surgical resection by an experienced onco-pathologist. The tumours were classified according to the revised 2004 WHO classification of lung tumours [3]. All tumours were staged as per the tumour (T), nodes (N) and metastasis (M) staging system, American Joint Committee on Cancer, seventh edition. Evaluation of driver mutation status was done by real-time polymerase chain reaction for epidermal growth factor receptor (EGFR), immunohistochemistry for Anaplastic lymphoma kinase (ALK), ROS proto-oncogene 1 (ROS1) and PD-L1. Next-generation sequencing was done in some patients. Patients were managed as per the decision taken in the multidisciplinary thoracic disease management group meeting. The choice of chemotherapy was decided by the treating physician. Patients who failed to follow-up were contacted by telephone to assess their status and survival. Patients for whom no medical records were available were excluded from the study.

### Clinical outcomes

In patients with metastatic disease on palliative therapy, response assessment was done by imaging every 2–3 months or at the development of clinical features of progression and was classified according to the Response Evaluation Criteria in Solid Tumors version 1.1 [21]. The progression-free survival (PFS) was defined as the interval from the start of systemic therapy to the date of progression or death due to any cause before disease progression. The patients who were lost to follow-up were censored on the date of their last follow-up. The overall survival (OS) was defined as the interval from the date of diagnosis to the date of death. Patients who were alive at the end of the study were censored at the date of the last follow-up.

### Statistical analysis

As this was a retrospective study of a rare tumour, the sample size was not calculated. We included all patients fulfilling the eligibility criteria during the study period. All statistical analyses were done using the SPSS software (Version 23, IBM Corp, Armonk, NY, USA), and the Kaplan–Meier curve was generated using R software (Vienna, Austria). The categorical variables were summarised by frequency and percentage and the continuous variables by median and range. Survival analysis was done using the Kaplan–Meier method [22] and the hazard ratio (HR) was calculated using the Cox proportional model [23, 24]. Univariate and multivariate analyses were performed to compare the OS to various factors using the log-rank test and Cox regression analysis, respectively. All *p* values were two-sided with a 95% confidence interval (95% CI) level. A *p*-value of less than 0.05 was considered statistically significant.

## Results

### Baseline patient characteristics

**Figure 1.**
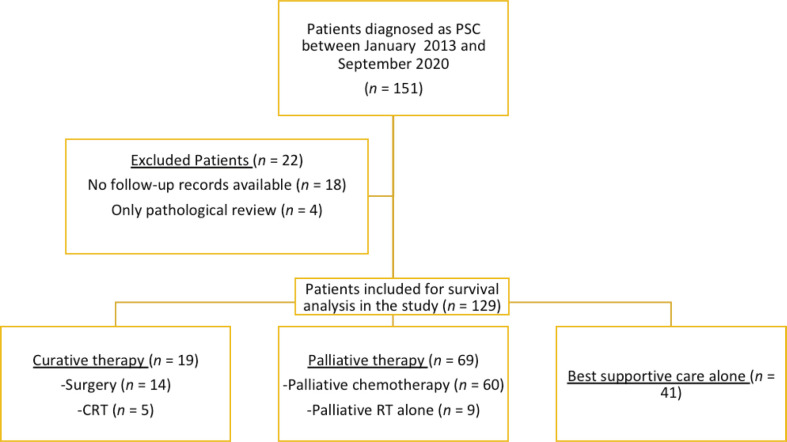
Patient flow diagram, showing the details of included and excluded patients. PSC, Pulmonary sarcomatoid carcinoma; CRT, Concurrent chemotherapy and radiotherapy.

A total of 151 patients were diagnosed with PSC during the period between January 2013 and September 2020 (Figure 1). Follow-up data were available for 129 patients and were included in the analysis. The baseline demographic, clinicopathological and imaging characteristics are summarised in Table 1. The median age was 63 years (range, 18–87). Almost half the patients presented with poor performance status (PS) of 2–4.

### Disease-related characteristics

Eighty-six patients (66.7%) had metastatic cancer at the time of diagnosis. A majority of patients had multiple sites of metastasis including lung in 45 (34.9%), pleura in 35 (27.1%), bone in 30 (23.3%), adrenal in 29 (22.5%), brain metastases in 27 (20.9%) and non-regional lymph node in 24 (18.6%). Baseline PET-CT scan data was available in 71 (47%) patients, which revealed a high maximum standardized uptake value (SUVmax) in the majority of the patients. The median SUVmax was 20.2 (range, 5–45.7). Pleomorphic carcinoma was the predominant subtype of PSC, seen in about 88 (58%) patients.

### Molecular characteristics

The reports of EGFR mutation testing were available for 57 patients, out of which 4 (7%) patients had an EGFR mutation (two patients had exon 19 deletions, one had exon 21 L858R mutation and one had both exon 19 deletion and exon 21 L858R mutation). ALK reports were available in 40 cases [either Immunohistochemisty (IHC) or fluorescence in situ hybridisation (FISH)] and of these, two patients (5%) had ALK rearrangement. ROS1 rearrangement was not detected in any of the 40 patients who were tested. FISH for mesenchymal epithelial transition factor (MET) amplification was negative in all the six tested patients. Next-generation sequencing was performed in four patients which revealed TP53 mutation with EGFR amplification in one patient, Kirsten ras oncogene homolog (KRAS) exon 2 missense mutation (p.G12D) with PIK3CA (Phosphatidylinositol-4,5-Bisphosphate 3-Kinase Catalytic Subunit Alpha) exon 1 mutation in one, BRAF (B-Raf Proto-Oncogene, Serine/Threonine Kinase) V600E and KRAS exon 2 mutation (p.G12V) in one patient each.

### Treatment received

#### Surgery

Curative surgery was performed in 14 (10.9%) of the 129 evaluable patients. One patient had an R+ resection. One patient died in the immediate postoperative period due to aspiration pneumonia. The various treatment modalities are summarised in Table 2.

Post-operative staging as per the histopathological report according to the TNM classification was stage I in 3 (21.4%), stage II in 3 (21.4%) and stage III in 8 (57.1%) patients.

A total of six patients with locally advanced disease (three had an unresectable primary tumour and three had single station N2 on endobronchial ultrasonography) received neoadjuvant chemotherapy. Of these six patients, three subsequently underwent surgery; the remaining three patients developed progressive metastatic disease following neoadjuvant chemotherapy. Platinum-based doublet combination was used in five patients; one patient who had pulmonary blastoma received an ifosfamide and doxorubicin combination. The response rate to neoadjuvant chemotherapy is summarised in Table 3. One patient received neoadjuvant chemo-radiotherapy and had a partial response.

#### Adjuvant therapy

Nine patients received adjuvant chemotherapy. Three received adjuvant postoperative radiotherapy (two due to N2 disease, one due to a positive margin). Platinum-based doublet combination was the most common regimen used in the adjuvant setting.

#### Radical radiotherapy

Five patients received radical intent radiotherapy with concomitant chemotherapy – indication being N2/N3 disease in three patients and unfit for surgery in two patients. Weekly paclitaxel 80 mg/m^2^ and carboplatin area under curve 2 mg/mL/minute were used as the concurrent chemotherapy regimen in all patients. One patient received stereotactic body radiation therapy to the lung as the primary treatment modality because of poor lung function.

#### Palliative therapy

Of the 129 evaluable patients, palliative intent systemic therapy was given to 66 patients (51.2%), of which six patients received therapy post-progression on curative intent therapy. The systemic therapy regimens used were platinum-based chemotherapy in 50 (75.8%), non-platinum based in 9 (13.6), tyrosine kinase inhibitors in 5 (7.6%) and ICIs in 2 (3.0%). Four patients had an EGFR mutation, of which two patients received targeted treatment with gefitinib; one patient died due to sepsis and another patient defaulted before any targeted therapy could be given. The PFS for the two patients who received EGFR tyrosine kinase inhibitors (TKI) was 5 and 11 months. One patient had ALK rearrangement and received crizotinib in the first line; this patient progressed on crizotinib within 2 months. Two patients who received erlotinib on a compassionate basis in the first-line setting due to poor PS went on to receive second-line chemotherapy. One patient who had metastatic disease with BRAF V600E mutation was treated with only palliative chemotherapy as BRAF inhibitor was not feasible. Second-line chemotherapy could be administered in 15 (22.7%) patients. Gemcitabine was the most frequently used second-line agent. Only about 6 (9.1%) patients could receive third-line therapy. The response rates to the different lines of palliative chemotherapy are summarised in Table 4. The objective response rate (ORR) for first, second and third-line chemotherapy was 15.2%, 13.3% and 0%, respectively. Two patients received ICIs in the first line with pembrolizumab and the PFS were 4 and 8 months. One patient who received ICIs in the second-line setting with nivolumab had a PFS of 3 months. Forty-one (31.8%) patients were unfit for any form of therapy due to poor PS. These patients have been advised the best supportive care (BSC) alone.

### Survival outcomes

**Figure 2.**
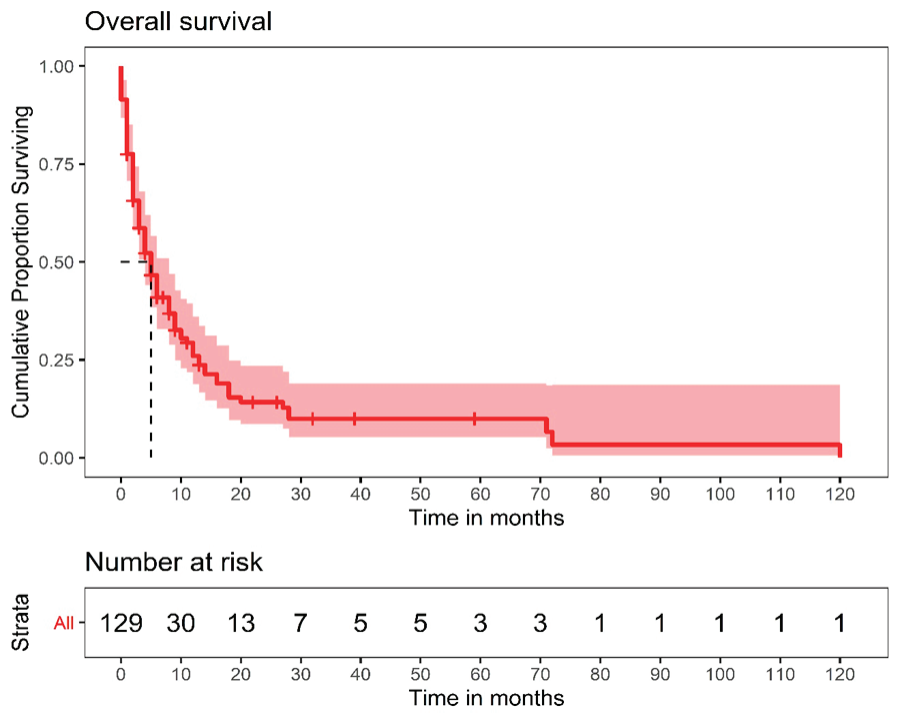
OS of the entire patient cohort.

One hundred and twenty-nine patients were included in the survival analysis (Figure 1). The median follow-up duration for the whole cohort was 32 months (range, 15.0–48.9). The median OS for the entire patient cohort was 5 months (95% CI, 3.4–6.5) (Figure 2). The median OS of patients who received curative-intent treatment (surgery or concurrent chemo-radiotherapy (CTRT)) was 14 months (95% CI: 6.5–21.4) and the corresponding 1- and 5-year survivals were 56% (Standard error (SE): 11.1) and 23% (SE: 9.9), respectively. The median OS for patients treated with palliative intent was 4 months (95% CI: 2.6–5.3, *p* = 0.00052) (Figure 2), and the corresponding 1- and 5-year survival rates were 20% (SE: 4.5) and 6% (SE: 3.2), respectively. The median OS according to the various treatment modalities is summarised in Table 5. For patients who received palliative treatment for locally advanced or metastatic disease, the median PFS for first-line chemotherapy was 4 months (95% CI: 2.95–5.04), second-line 4 months (95% CI: 0.33–7.67) and third-line 1 month (95% CI 0.21–1.78)

### Factors affecting survival

The median OS for patients according to the stage of the disease was 28 months (95% CI: 0–71.20 months) for stage I, 9 months (95% CI: 0–18.80) for stage II, 6 months (95% CI: 2.85–9.14) for stage III and 4 months (95% CI: 2.83–5.16) for stage IV (HR: 1.52, 95% CI: 1.12–2.06), *p* = 0.007. The corresponding 2-year survival rates were 66.7% (SE: 27.2) for patients with stage I, 25% (SE: 21.7) for II, 17% (SE: 7.0) for stage III and 9% (SE: 4.1) for stage IV disease.

The factors significantly affecting the survival outcomes in the univariate analysis were Eastern Cooperative Oncology Group PS (ECOG PS), the intent of treatment, stage of the disease, T stage, M stage, presence of brain metastasis and the type of initial treatment received. On multivariate analysis, only brain metastasis and administration of palliative chemotherapy were found to be significant. The univariate and multivariate analyses are summarised in Supplementary Table 1.

## Discussion

Our study represents one of the largest single-institution case series of patients with PSC.

In our study, the majority of the patients were smokers (70.6%), which is similar to that reported in other studies [25]. However, in an earlier study from our group, we found that 52% of patients with NSCLC were non-smokers; thus, smoking appears to be more common in patients with PSC than NSCLC in our patients [26]. However, we found that smoking did not significantly influence the survival of our patients with PSC. PSC was also seen more frequently in males than females (81.5% in males) [2, 11, 27]. The majority of patients who had metastatic disease had poor PS and the proportion of patients who received first-line or subsequent lines of therapy was low, which reflects the aggressive disease biology.

We found that the median SUV max of the tumour on the baseline PET-CT was 20.2, which is similar to that reported in other studies which reported that PSC has intense SUV uptake values [28, 29]. This suggests that PSCs are F-fluorodeoxyglucose (FDG)-avid tumours and that performing a PET-CT scan would be an appropriate staging test, especially in patients who are being planned for radical therapy. The median tumour size was 7.5 cm and the majority presented with advanced T stages (about 91.4 % were of T3 and T4). In a similar study of 51 cases of PSC, the median T size was 6 cm and the incidence of T3/T4 disease was 60.7%. However, this frequency was relatively higher in our study when compared with case series from other countries. Interestingly, only three patients had T1 disease in our study. Similarly, the percentage of stage III and IV disease was 95.3%, which was markedly higher in contrast with other case series [10, 30].

Most studies on PSC have reported that PSC carries a poor prognosis with the caveat that these are small case series or single-institution studies. In one study from the Mayo Clinic, a matched comparison between PSC and typical NSCLC demonstrated that PSC histology was associated with significantly poorer survival than other NSCLC histologies. In this study, the median OS was 9.9, 25.2 and 16.8 months for PSC, adenocarcinoma and squamous cell carcinoma, respectively [9]. Similarly, a study from the Surveillance, Epidemiology and End Results database on PSC also revealed a significantly worse OS for patients with PSC compared with matched NSCLC controls [31]. The survival in our study was markedly lower when compared to other published case series which had reported a median survival range of 10–19 months. The poor survival in our study, when compared to the other case series, may be attributed to a multitude of causes. A high percentage of patients presented with a very advanced stage, with early-stage disease (stage I and II) accounting for only 4.7%. The percentage of patients who could receive curative-intent therapy was also markedly lower in comparison with other reported case series: surgery in 9.3% and CTRT in 3.3% of patients [9, 31]. Other reasons include a higher percentage of patients with poor PS, high nodal stage, higher incidence of brain metastasis, higher rates of upfront BSC, lower incidence of targetable driver mutation and the lower use of ICIs.

There are conflicting reports on the prevalence of EGFR mutation in sarcomatoid carcinoma of the lung. In a study by Jiang *et al* [32], the EGFR mutation was seen in 9 out of 32 patients (28.1%). In another study of 22 patients by Italiano *et al* [33], none had EGFR mutations. Likewise, the efficacy of EGFR TKIs in PSC is unknown. Few reports have suggested that PSC with EGFR mutations respond poorly to TKIs [32, 34]. In our study, the frequency of EGFR mutation was 7% (4/57 patients) and two patients had a reasonable response to EGFR TKI. The prevalence of EGFR mutation in lung sarcomatoid carcinoma is lower than that reported in our patients with adenocarcinoma but similar to that reported in our patients with squamous cell lung cancer [35]. Similarly, the prevalence of ALK rearrangement in PSC is not clear [36]. Chen *et al* [37], in a study of 82 patients with PSC, found that ALK rearrangement was seen in three patients (3.6%). Gelibter *et al* [38] reported a case of PSC with ALK rearrangement who had a PFS of 15 months with alectinib. Two patients in our study had ALK rearrangement by IHC and the only patient who received crizotinib had a poor response with a PFS of 3 months.

PSC was historically considered to be resistant to chemotherapy [39–42]. Our study also revealed a poor response of PSC to chemotherapy. The response rates to first-line chemotherapy in our study were 15.2%, 13.3% for the second line, and 0% for the third line. This is similar to a study by Vieira *et al* [15], in which platinum-based chemotherapy was given to 73% of patients (*n* = 97), the response rate to chemotherapy was just 16.5% and about 69% of the patients had progressive disease at the time of first evaluation after the initiation of chemotherapy. These rates are much higher when compared with conventional NSCLC. For instance, in a combined analysis of three randomised controlled trials from the Southwest Oncology Group, the response rate to first-line platinum-based chemotherapy was 27% and the progression rate was 38% for NSCLC [43]. Molecular analysis of PSC has revealed a high frequency of mutation in certain genes like TP53 in 60%–80% [20, 44] and KRAS in 30%–35% of the cases [44, 45] and this may be the reason for resistance to chemotherapy [46]. The role of antiangiogenic therapy has been explored in the treatment of PSC. Apatinib, an antiangiogenic TKI which targets the vascular endothelial growth factor receptor 2 (VEGFR2) has been reported to cause a sustained response of 14 months in a case report by LI *et al* [47]. TP53 mutation has been postulated to be a predictive biomarker of response to antiangiogenic agents [48, 49]. Since these tumours have a high prevalence of TP53 mutations, further studies are required to evaluate the role of VEGF targeting agents.

Very little progress has been made in the treatment of PSC to date. Given the high incidence of KRAS and MET mutation in PSC and with the arrival of novel drugs to target these mutations [50–52], the survival is expected to improve in the future [53, 54]. Bringing down the cost of ICIs would go a long way to improve the outcomes in lower-middle-income countries like India [55]. Ongoing studies in recurrent or metastatic PSC are evaluating the role of dual ICIs like nivolumab + ipilimumab (NCT02834013), durvalumab + tremelimumab (NCT03022500), chemotherapy combined with ICIs (ifosfamide + doxorubicin + durvalumab, NCT04224337), the combination of chemotherapy, ICIs and anti-VEGF agents (toripalimab + bevacizumab + nab-paclitaxel and carboplatin, NCT04725448) and savolitinib in MET exon 14 mutated PSC (NCT02897479).

The limitations of our study include the retrospective nature subject to an inherent bias, a lack of central pathological review and the heterogeneous nature of the chemotherapies used. We also were limited in our ability to perform extensive molecular testing in all patients; hence, we do not have information on the molecular profiles of all the tumours.

## Conclusion

PSC presents in advanced stages, has a poor response to the current chemotherapeutic drugs, and is associated with poor survival outcomes. Further studies using molecular characterisation, ICIs and targeted therapy will be indispensable to improve the survival of these patients.

## Conflicts of interest

There are no conflicts of interest.

## Figures and Tables

**Supplementary Table 1.** Univariate and multivariate analyses of factors associated with OS.

		Univariate analysis			Multivariate analysis	
Characteristics	*N* (%)	Median OS (months; 95% CI)	*p-*value	HR (95% CI)	*p-*value	HR (95% CI)
Age (years) ≤65 >65	54 75	5 (3.7–6.2) 4 (1.8–6.1)	0.39	1.19 (0.79–1.77)		
ECOG PS 0–1 2–4	61 68	9 (6.5–11.4) 2 (1.2–2.7)	0.001	1.74 (1.41–2.15)	0.92	1.15 (0.64–2.07)
Smoker Yes No	89 37	5 (2.3–7.6) 4 (2.6–5.3)	0.56	0.88 (0.57–1.36)		
Sex Male Female	106 23	4 (2.0–5.9) 8 (2.3–13.6)	0.23	0.73 (0.43-1.22)		
Comorbidities Yes No	46 83	4 (0.5–7.4) 5 (3.2–.7)	0.18	1.31 (0.87–1.97)		
Stage I II III IV	3 4 35 87	28 (0–71.2) 9 (0–18.8) 6 (2.8–9.1) 3 (1.5–4.4)	0.007	1.53 (1.12–2.08)	0.51	0.82 (0.35–1.91)
T stage 1–2 3–4	11 113	9 (0–18.7) 5 (3.6–6.3)	0.03	1.40 (1.03–1.96)	0.507	1.22 (0.83–1.79)
N stage 0–1 2–3	33 91	6 (4.1–7.8) 4 (1.8–6.1)	0.44	1.18 (0.76–1.84)		
M stage 0 1	34 86	8 (4.6–11.2) 4 (2.4–5.5)	0.014	0.76 (0.61–0.94)	0.30	0.89 (0.30–2.66)
Brain metastasis Yes No	27 102	1 (0.3–1.6) 6 (3.5–8.4)	0.001	4.2 (2.54–7.08)	0.018	2.47 (1.35–4.49)
Curative surgery Done Not done	14 115	18 (2.5–33.4) 4 (2.5–5.4)	0.000		0.926	1.08 (0.19–6.19)
Systemic chemotherapy Given Not given	66 50	8 (5.1–10.8) 2 (1.4–2.5)	0.000		0.037	2.2 (1.04–4.94)

PS, Performance status; OS, Overall survival; HR, Hazard ratio; CTRT, Concurrent chemo-radiotherapy; RT, Radiotherapy; BSC, Best supportive care

**Figure 3.**
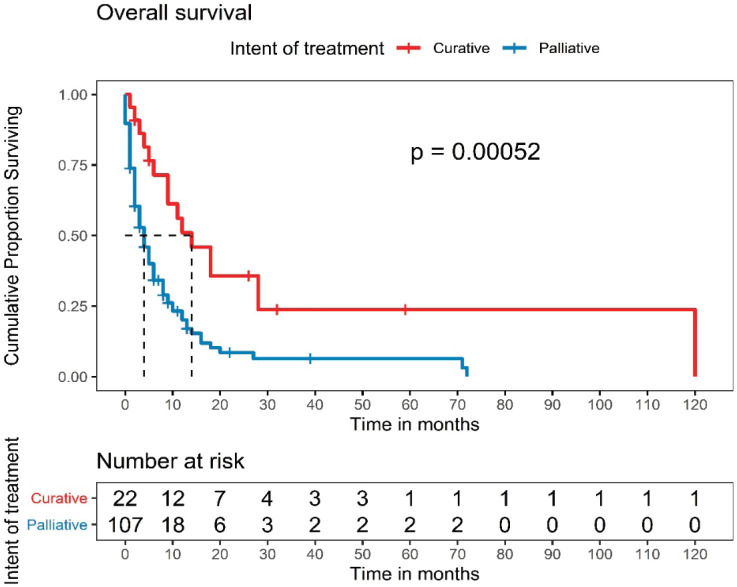
OS of patients according to the intent of treatment.

**Table 1.** Demographic and tumour characteristics.

Characteristics	Results, in number (percentage)
Median age in years (*N* = 129) Age ≤ 65 years Age > 65 years	63 (18–87) 54 (41.9) 75 (58.1)
Sex (*N* = 129) Male Female	106 (82.2) 23 (17.8)
Smoking status (*n* = 126) Current/former smoker Nonsmoker	89 (69.0) 37 (28.7)
Comorbidities (*n* = 129) Present^a^ None	46 (35.7) 83 (64.3)
Family history of cancer (*n* = 129) Yes No	3 (2.3) 126 (97.7)
ECOG PS (*n* = 129) 0 1 2 3 4	4 (3.1) 57 (44.2) 39 (30.2) 20 (15.5) 9 (7.0)
Presenting symptom (*n* = 128) Cough Chest pain Dyspnoea Bone pain Others	68 (53.1) 32 (24.8) 5 (3.9) 6 (4.7) 17 (13.3)
Tumour size in cm (*n* = 123) Mean ± SD Median	7.8 ± 2.94 7.7 cm (1.7–20)
Tumour stage (*n* = 124) T1 T2 T3 T4	1 (1.6) 9 (7.3) 20 (16.1) 93 (75)
Nodal stage (*n* = 124) N0 N1 N2 N3	28 (22.6) 5 (4.0) 39 (31.5) 52 (41.9)
Metastatic stage (*n* = 129) M0 M1	43 (33.3) 86 (66.7)
Stage of disease at diagnosis (*n* = 129) 1 2 3 4	3 (2.03) 4 (3.1) 35 (27.1) 87 (67.4)
Histopathological type (*N* = 151) Pleomorphic carcinoma Giant cell carcinoma Spindle cell carcinoma Carcinosarcoma Pulmonary blastoma Sarcomatoid carcinoma (not subtyped)	88 (58.3) 8 (5.3) 8 (5.3) 3 (2.0) 4 (2.6) 40 (26.5)

ECOG, Eastern Cooperative Oncology Group; SD, Standard deviation

a Comorbidities include hypertension, diabetes, chronic obstructive pulmonary disease, coronary artery disease, tuberculosis, cerebrovascular disease, chronic kidney disease, etc.

**Table 2.** Treatment modalities.

Treatment	All patients in absolute numbers (percentage)
Initial treatment modality (*n* = 129) Surgery ± adjuvant therapy CRT Palliative chemotherapy/targeted therapy Radiotherapy alone Upfront BSC	14 (10.9) 5 (3.9) 60 (46.5) 9 (7.0) 41 (31.8)
Surgery (*n* = 129) Yes No	14 (10.9) 115 (89.1)
Type of surgery (*n* = 14) Wedge resection Lobectomy Bilobectomy Pneumonectomy	2 (14.2) 10 (71.4) 1 (7.1) 1 (7.1)
Neoadjuvant therapy (*n* = 7) NACT NACTRT	6 (85.8) 1 (14.2)
Adjuvant therapy (*n* = 14) No adjuvant therapy Adjuvant chemotherapy alone Adjuvant chemotherapy + PORT	5 (35.7) 6 (42.8) 3 (21.4)

NACT, Neoadjuvant chemotherapy; NACTRT, Neoadjuvant chemotherapy + concurrent radiotherapy; PORT, Post-operative radiotherapy; CTRT, Concurrent chemo-radiotherapy; BSC, Best supportive care. Patients with poor PS are considered for BSC

**Table 3.** Neoadjuvant chemotherapy regimens and the response to chemotherapy.

Patient No.	Neoadjuvant chemotherapy regimen	Histological type	Best response	Surgery
1	Cisplatin + vinorelbine	Pleomorphic carcinoma	Stable disease	Yes
2	Cisplatin + pemetrexed	Pleomorphic carcinoma	Partial response	Yes
3	Ifosfamide + doxorubicin	Pulmonary blastoma	Partial response	Yes
4	Paclitaxel + carboplatin	Spindle cell carcinoma	Progressive disease	No
5	Pemetrexed + carboplatin	Spindle cell carcinoma	Progressive disease	No
6	Paclitaxel + carboplatin	Pleomorphic carcinoma	Progressive disease	No

**Table 4.** Response rate to subsequent lines of chemotherapy.

Palliative chemotherapy	N	Complete response	Partial response	Stable disease	Progressive disease	ORR in %
First line, (*n* = 66) Platinum-based Ifosfamide + doxorubicin Taxanes ICIs Single-agent gemcitabine Crizotinib Gefitinib (EGFR mutant) Gefitinib (compassionate basis)	50 4 2 2 3 1 2 2	0 0 0 0 0 0 0 0	9 0 0 1 0 0 0 0	20 3 1 1 2 0 1 2	21 1 1 0 1 1 1 0	18 0 0 50 0 0 0 0
Second line *n* = 15 Gemcitabine-based Taxanes Pemetrexed ICIs Pazopanib	7 3 3 1 1	0 0 0 0 0	1 0 1 0 0	4 1 2 0 1	2 2 0 1 0	14.2 0 33.3 0 0
Third line *n* = 6 Taxanes Doxorubicin + ifosfamide Gemcitabine	4 1 1	0 0 0	0 0 0	0 0 0	4 1 1	0 0 0

EGFR, Epidermal growth factor receptor; ORR, Objective response rate

**Table 5.** Outcomes for the various treatment modalities in patients with PSC.

Treatment modality	Median OS	1-year survival rate	5-year survival rate
Entire cohort (*N* = 129)	5 months (95% CI: 3.40–6.50)	26% (S.E: 4.3)	10% (S.E: 3.3)
Curative intent (*n* = 22)	14 months (95% CI: 6.50–21.40)	56% (S.E: 11.1)	23% (S.E: 9.9)
Palliative intent (*n* = 107)	4 months (95% CI: 2.60–5.30)	20% (S.E: 4.5)	6% (S.E: 3.2)
Curative surgery (*n* = 14)	18 months (95% CI: 2.59–33.4)	64% (S.E: 12.8)	33% (S.E: 13.1)
Concurrent chemoradiation (*n* = 5)	11 months (95% CI: 2.99–19.00)	33% (S.E: 27.2)	0%
Palliative radiotherapy (*n* = 9)	4 months (95% CI: 1.91–6.08)	19% (S.E: 17.2)	0%
Palliative chemotherapy (*n =* 60)	8 months (95% CI: 5.24–10.75)	37.5% (S.E: 7.0)	6% (S.E: 5.0)
BSC (*n* = 41)	1 month (95% CI: 0.43–1.57)	3.4% (S.E: 3.2)	0%
